# Global changes in the proteome of *Cupriavidus necator* H16 during poly-(3-hydroxybutyrate) synthesis from various biodiesel by-product substrates

**DOI:** 10.1186/s13568-016-0206-z

**Published:** 2016-05-17

**Authors:** Parveen K. Sharma, Jilagamazhi Fu, Victor Spicer, Oleg V. Krokhin, Nazim Cicek, Richard Sparling, David B. Levin

**Affiliations:** Department of Biosystems Engineering, University of Manitoba, Winnipeg, MB R3T 5V6 Canada; Department of Physics and Astronomy, University of Manitoba, Winnipeg, Canada; Manitoba Centre for Proteomics and Systems Biology, University of Manitoba, Winnipeg, Canada; Department of Microbiology, University of Manitoba, Winnipeg, MB R3T 2N2 Canada

**Keywords:** Short chain length polyhydroxyalkanoates, scl-PHAs, Poly(3-hydroxybutyrate), Biodiesel-derived glycerol, Biodiesel-derived glycerol bottom, Biodiesel-derived free fatty acids, Proteomes, Metabolism, β-oxidation

## Abstract

Synthesis of poly-[3-hydroxybutyrate] (PHB) by *Cupriavidus necator* H16 in batch cultures was evaluated using three biodiesel-derived by-products as the sole carbon sources: waste glycerol (REG-80, refined to 80 % purity with negligible free fatty acids); glycerol bottom (REG-GB, with up to 65 % glycerol and 35 % free fatty acids), and free fatty acids (REG-FFA, with up to 75 % FFA and no glycerol). All the three substrates supported growth and PHB production by *C. necator*, with polymer accumulation ranging from 9 to 84 % cell dry weight (cdw), depending on the carbon source. To help understand these differences, proteomic analysis indicated that although *C. necator* H16 was able to accumulate PHB during growth on all three biodiesel by-products, no changes in the levels of PHB synthesis enzymes were observed. However, significant changes in the levels of expression were observed for two Phasin proteins involved with PHB accumulation, and for a number of gene products in the fatty acid β-oxidation pathway, the Glyoxylate Shunt, and the hydrogen (H_2_) synthesis pathways in *C. necator* cells cultured with different substrates. The glycerol transport protein (GlpF) was induced in REG-GB and REG-80 glycerol cultures only. *Cupriavidus necator* cells cultured with REG-GB and REG-FFA showed up-regulation of β-oxidation and Glyoxylate Shunt pathways proteins at 24 h pi, but H_2_ synthesis pathways enzymes were significantly down-regulated, compared with cells cultured with waste glycerol. Our data confirmed earlier observations of constitutive expression of PHB synthesis proteins, but further suggested that *C. necator* H16 cells growing on biodiesel-derived glycerol were under oxidative stress.

**Electronic supplementary material:**

The online version of this article (doi:10.1186/s13568-016-0206-z) contains supplementary material, which is available to authorized users.

## Introduction

An increasing demand for renewable, bio-based products is driving the global market for bioplastics. Polyhydroxyalkanoates (PHAs) are natural polyesters synthesized by bacteria as carbon and energy storage molecules, and accumulate intra-cellularly (Anderson and Dawes 1990; Sudesh et al. 2000; Rehm 2010; Gao et al. 2011) under nutrient-limiting conditions in the presence of excess carbon. Limiting concentrations of oxygen, nitrogen, phosphate, and sulphur, or trace element ions like magnesium, calcium, and iron, have been shown to induce synthesis of PHAs (Lee 1996; Kessler and Witholt 2001).

PHAs are biodegradable, insoluble in water, nontoxic, biocompatible, piezoelectric, thermoplastic and/or elastomeric. These features make PHAs suitable for several applications in the packaging industry, medicine, pharmacy, agriculture, and food industry, as raw materials for the production of enantiomerically pure chemicals, and for the production of paints. PHAs are grouped into two main categories: short chain length PHAs (scl-PHAs), having carbon chains of C_4_-C_5_, and medium chain length PHAs (mcl-PHAs), which have carbon chains of C_6_–C_14_ (Khanna and Srivastava 2005).

Polyhydroxybutyrate (PHB), a polymer of 3-hydroxybutyrate, is a highly biodegradable natural polyester polymer produced for various applications. PHBs have been produced commercially, but their high production costs make them economically unattractive compared to petroleum-based plastics. Carbon substrates used for PHB synthesis account for 40–48 % of the production costs (Choi and Lee 1997) and there is now a global effort to decrease production costs by using low cost carbon substrates (Cavalheiro et al. 2009; Obruca et al. 2010; Povolo et al. 2010; Budde et al. 2011; Ashby et al. 2005; Solaiman et al. 2006).

Biodiesel is made through a chemical process called trans-esterification from animal fat and vegetable oils. The process generates two products, fatty acid alkyl esters, the chemical name for biodiesel, and glycerol (a valuable byproduct usually sold for soaps and other products). Ten (10) tons of glycerol is produced for every 100 tons of biodiesel produced. Some biodiesel-derived glycerol is refined to high purity for commercial applications, but this is a costly process (da Silva et al. 2009). Disposal of the waste crude glycerol in municipal sewage systems is prohibited in many jurisdictions, or is associated with a disposal fee. Moreover, it is not economically feasible for small biodiesel plants to purify glycerol. Thus, impurities such as free fatty acids, methanol, heavy metals and salts (NaCl) in crude glycerol render biodiesel-derived glycerol as a “waste” product (Chatzifragkou and Papanikolaou 2012; Vandamme and Coenye 2004; Fu et al. 2015). Impurities like free fatty acids in biodiesel-derived glycerol, however, may provide an additional carbon source for microbial growth and PHA production.

*Cupriavidus necator* H16 (also known as *Ralstonia eutropha* H16), a widely used bacterium for PHB production, can accumulate up to 85 % of cell biomass as PHB under nutrient-limiting conditions (Vandamme and Coenye 2004). *Cupriavidus necator* can utilize a wide variety of carbon substrates like starch and lipids (Almeida et al. 2012; Mazur et al. 2009; Mifune et al. 2010). The most commonly used substrates for the fermentative production of scl-PHA are sugars. However, agricultural residues have also been tested in recent years (Morais et al. 2014; Escapa et al. 2013).

Biodiesel-derived waste materials represent complex mixtures of carbon sources, which may effect microbial metabolism in different ways. PHB synthesis by *C. necator* strains using biodiesel-derived glycerol as a sole carbon source has been reported (Koller et al. 2005; Zhu et al. 2013) and crude glycerol bottom (a mixture of glycerol and fatty acids) or free fatty acids purified from biodiesel-diesel derived glycerol bottom, have been investigated for mcl-PHA production (Fu et al. 2015). Although salts like NaCl or K_2_SO_4_ present in some biodiesel byproducts have been reported to effect PHB synthesis (Peplinski et al. 2010; Lee et al. 2009), growth and PHB synthesis by *C. necator* were not affected by impurities present in biodiesel-derived glycerol (Cavalheiro et al. 2009). Similar observations have been made for growth and PHA synthesis by *Pseudomonas putida* KT2440 (Escapa et al. 2013) and *P. putida* LS46 for mcl-PHA synthesis (Fu et al. 2014).

Previous studies have investigated changes in gene and gene product expression in *C. necator* H16 (=*R. eutropha* H16) cultured under different growth conditions (Peplinski et al. 2010; Lee et al. 2009; Raberg et al. 2011; Schwartz et al. 2009; Ibrahim and Steinbüchel 2009; Potter and Steinbuchel 2005; Jendrossek and Pfeiffer 2014). However, global changes in protein expression profiles in *C. necator* H16 cultured with biodiesel production by-products (biodiesel-derived glycerol bottoms, semi-purified glycerol, and free fatty acids) have not been previously reported. We conducted one dimensional (1D) liquid chromatography followed by mass spectroscopy (LC/MS/MS) analysis to evaluate changes in protein expression levels of key metabolic pathways related to growth and PHB synthesis using these biodiesel by-product streams.

## Materials and methods

### Organism, media, and cultivation

*Cupriavidus necator* strain H16 DSM428 (equivalent to *C. necator* ATCC 17699 and *Ralstonia eutropha* H16) was procured from DSMZ, Germany. Cultures were reconstituted in Luria broth as described by DSMZ and streaked on LB plates for a single colony isolates. Single colony isolates were grown in Luria broth and preserved as glycerol stocks at −80 °C. Experiments were carried out in 500 mL baffled flasks containing 100 mL Ramsay’s minimal medium (RMM) (Ramsay et al. 1992) consisting of 6.7 g/L Na_2_HPO_4_·2H_2_O, 1.5 g/L KH_2_PO_4_, 1 g/L (NH4)_2_SO_4_, 0.2 g/L MgSO_4_·7H_2_O, 0.01 g/L CaCl_2_·2H_2_O, 0.06 g/L Fe(NH_4_)_2_(citrate)_2_, and 1 mL/L of trace element solution (0.3 g/L H_3_BO_3_, 0.2 g/L CoCl_2_, 0.1 g/L ZnSO_4_·7H_2_O, 0.03 g/L MnCl_2_·4H_2_O, 0.02 g/L NaMoO_4_·2H_2_O, 0.02 g/L NiCl_2_·6H_2_O, and 0.01 g/L CuSO_4_·5H_2_O).

PHB synthesis by *C. necator* H16 was investigated using biodiesel-derived waste products procured from Renewable Energy Group (REG), Seneca, IL USA. The substrates used were 2 % v/v biodiesel-derived glycerol (REG-80, a commercial glycerol from biodiesel industry containing on an average 85 % glycerol), 2.0 % w/v REG-glycerol bottoms (REG-GB), and 1 % v/v REG-free fatty acids (REG-FFA). The compositions of the three biodiesel-derived substrates are presented in Table 1. The pH of the medium was adjusted to 7.0. The flasks were incubated at 30 °C on rotary shaker up to 120 h.

**Table 1:** Composition of REG-glycerol, REG-fatty acids and REG-glycerin bottom

Component (%)	REG-80 (glycerol)	REG-FFA (free fatty acids)	REG-GB (glycerol bottom)
Glycerin	78 (82)^a^	ND	45–65 (54)
Potassium acetate	<15 (10)	ND	ND
Moisture/volatiles	<13 (11)	<6	2 [0 (0.5)]
MONG^a^/fatty acids	0.02 (1.0)	>60 (75)	20–35 (25)
Methyl esters	0.05 (0.3)	10–15	–
Methanol	–	<1	0.25
Ash	–	–	20 (13)
Density (Kg/m^3^)	1.25 (1.23)	0.74–0.77 (7.5)	–
Insoluble impurities	–	<2	–
Color	Brown	Brown	Black
Energy content (Btu/lb)	5000–10,000 (7000)	12,000–14,000 (13,000)	ND

*ND* not determined (Provided by Renewable Energy Group, Saneca, IL)

^a^Figures in parenthesis are average values

Inocula for batch experiments in flasks were prepared by picking a single colony of *C. necator* H16 from a streaked plate and inoculating Luria Bertini (LB) broth in glass tubes. Pre-experiment inocula cultures were incubated for 18 h at 30 °C. Experimental flasks were inoculated with 5 % of the experimental culture volume, incubated at 30 °C for 72 h, and then analyzed for cell biomass and PHB production. All experiments were conducted with three independent biological replicates.

### Cell growth measurement

Samples (40 mL) of each culture, taken at 24, 48, 72, 96 and 120 h post inoculation (h pi) and centrifuged at 4500×*g* for 20 min in a Sorvall RC6-Plus centrifuge. The pellets were washed twice in 0.9 % NaCl and dried at 60 °C for 48 h to estimate cell dry mass as described earlier (Sharma et al. 2012).

### Analysis of residual glycerol, free fatty acids, and ammonium nitrogen

Residual glycerol was estimated in REG-80, REG-GB, and REG-FFA containing culture media by ion chromatography using a Dionex ICS-3000 system fitted with a CarboPac PAI 4 × 250 mm column, with 0.05 mmol/L H_2_SO_4_ as the eluent, and a refractive index detector. Residual fatty acids in REG-80, REG-GB, REG-FFA containing cultures were estimated by as gas chromatography (GC) analysis. Five (5) g of REG-80, REG-GB, and REG-FFA were suspended in 5 mL water, mixed with 5 mL chloroform, and then mixed by vortexing. The mixture was allowed to stand overnight, after which 1 mL of the chloroform layer (bottom layer) was removed by pipette and mixed with 2 mL methanol containing 15 % sulphuric acid and 1 mL chloroform containing 0.5 mg/mL heptadecanoic acid. The mixture was heated at 100 °C in screw-capped tube. After 5 h, 1 mL water was added and the mixture was allowed to stand overnight. The lower layer containing fatty acid methyl esters was analyzed by gas chromatography (GC, Agilent 7890A) fitted with a flame ionization detector (FID) and DB-23 capillary column (30 mCell growth measurement × 320 × 0.25 µm; Agilent, California, USA). Samples (1 µl) were injected (using a CTC auto sampler) into the FID with a split ratio at 1:10. Helium was used as carrier gas at a flow rate of 1.78 mL/min. The injector and oven temperatures were 260 °C. The ramping program for the oven was as follows: initial temperature of 60 °C for 4 min; followed by an increase from 60 to 250 °C, with incremental increases of 15 °C/min; followed by a final hold at 260 °C for 4 min. A Supelco 37 component FAME mix (Sigma-Aldrich) was used as standard for peak identification and calculation of response factors for different fatty acids. Fatty acid peaks were quantified by comparing the peak area of the internal standard (1 mg/mL benzoic acid), and corrected with its response factor. Total fatty acid content was achieved by adding the individual fatty acid concentration together. Ammonia nitrogen was measured using the Quikchem method 10-107-06-1-I for determination of ammonia in wastewater by flow injection analysis (Lachat Instrument, Colorado, USA), and the measured concentrations were rounded-off and reported to 2 significant digits.

### PHB quantification and monomer composition

Cultures were harvested by centrifugation at 4500×*g* for 30 min in a Sorvall RC6 Plus centrifuge. Cell pellets were dried for overnight at 60 °C and cell dry weights were determined. Dried samples were methylated as previously described (Braunegg et al. 1978) and was analyzed by gas chromatography (GC, Agilent 7890A) fitted with a flame ionization detector (FID) and DB-23 capillary column (30 m × 320 × 0.25 µm; Agilent, California, USA) as described earlier (Sharma et al. 2012). Molecular standards included 3-hydroxybutyrate and 3-hydroxyvalerate.

### Proteomic analyses

*Cupriavidus necator* H16 was cultured in RMM medium containing REG-GB (2 % w/v glycerol bottom), REG-80 (2 % v/v glycerol), and REG-FFA (1 % v/v REG free fatty acids). Samples (40 mL) were collected REG-GB at 24 h pi and these samples were designated as GB-24 (REG-glycerol bottom at 24 h pi). Samples were also taken from REG-80 cultures at 24 h pi and were designated as R80-24 (REG-glycerol at 24 h pi). Finally, samples were taken from REG-FFA cultures at 24 h pi and was designated as FFA-24 (REG-free fatty acids at 24 h pi). Samples were also taken form these cultures at 48 h pi and designated as GB-48, R80-48, and FFA-48, respectively.

All samples taken from the cultures were centrifuged at 4500×*g* for 10 min, and the resulting pellets were washed three times with phosphate-buffered saline (NaCl, 8 g/L; KCl, 0.2 g/L; Na_2_HPO_4_, 1.44 g/L; KH_2_PO_4_, 0.24 g/L, pH 7.4). Each treatment was carried-out with two biological replicates, and the 12 samples (6 conditions × 2 replicates) were frozen at −80 °C until protein extractions could be performed. Protein extracts from the 24 h pi samples for the REG-GB, REG-80, and REG-FFA grown cultures were prepared, processed, and subjected to 1D-LC–MS/MS analyses, as described below.

### Protein isolation, digestion, and peptide purification

#### Protein isolation

Frozen pellets were re-dissolved in 4 mL distilled-deionized (DI) water, of which 1 mL was used for total protein isolation. One mL of lysis Buffer (8 % of sodium dodecyl sulphate, 200 mM dithiothreitol, 200 mM Tris–HCl, pH 7.6) was added to each 1 mL sam ple of resuspended cell pellet and the solutions were mixed thoroughly by pipetting. The solutions were transferred to 10 mL Falcon tubes, which were then place in a boiling water bath for 5 min. After boiling, the samples were sonicated for 30 s, and then centrifuged at 16,000×*g* at room temperature. One mL of each supernatant was then transferred into a 1.5 mL Eppendorf tube.

#### Protein purification, digestion and peptide purification

One mL of total protein sample was transferred to an Amicon Ultra-15 10 K filter device (Millipore, Billerica, MA), followed by the purification, peptide digestion, and subsequent peptide purification steps described earlier (Verbeke et al. 2014), except that the trypsin: protein ratio used was 1:100 instead of 1:50. The purified peptides were lyophilized and re-dissolved in 0.1 % formic acid in water for subsequent 1D-LC–MS analysis.

#### Protein identification and quantification

Proteins were identified and quantified by 1D-LC–MS/MS as described by Gungormusler-Yilmaz et al. (2014), except that a specific peptide database derived from the *C. necator* H16 genome was used for alignment. Total ion count (TIC), which is the sum of all collision induced dissociation (CID) fragment intensities of member peptides were recorded.

### Proteomic data analysis

Protein expression trends were analyzed using our in-house lobe/UNITY analysis platform (Verbeke et al. 2014), which organizes proteins into annotation membership across four “higher-order variables” (HOVs) extracted from the IMG-ER “Export Gene Information” function (Markowitz et al. 2009). These HOVs were selected to both overlap somewhat and provide useful degrees of biological granularity, and include METACYC pathways, enzyme class (EC) numbers, clusters of orthologous group (COG) letters, and KEGG pathway modules.

The expression data obtained from *C. necator* H16 cells cultured with REG-GB, REG-80, and REG-FFA were analyzed as previously described (Riffat et al. 2015) for cross-state differences (from two independent biological replicates) on a protein-by-protein level: (Z0 = log2[TIC REG-FFA24-1]−log2[TIC REG80 24-1]; Z1 = log2[TIC REG-FFA24-2]−log2[TIC REG80 24-2]), and for intra-replicate variation (from two independent biological replicates) on a protein-by-protein level (R0 = log2[TIC REG-FFA 24-1]−log2[TIC REG-FFA 24-2]; R1 = log2[TIC REG80 24-1]−log2[TIC REG80 24-2]). Cross-state and intra-replicate comparisons were also made for proteomes derived from cultures with each of the other substrates: i.e. REG-GB vs REG-FFA and REG-GB vs REG-80 glycerol. The same cross-state and intra-replicate comparisons were preformed with samples collected at 48 h pi.

Cross-state populations exhibited standard deviations >5-times greater than their intra-replicate counterparts. This was a good initial indication of the quality of the experimental design and runs. These three different populations were each normalized (mean 0; standard-deviation 1) and subjected to a simple algorithm for merging them into a single normalized expression value called “Znet scores” (Markowitz et al. 2009), which were used in subsequent protein differential expression analyses. This final differential expression distribution was with the outermost 33 % of the population, with Znet-scores of N ± 1.0, the outermost 10 % of the population having absolute-value Znet-scores of N ± 1.65, and the outermost 5 % of the population having absolute-value Znet-scores of N ± 1.96. In our analyses, a positive (+) Znet score for any protein represents higher expression level in one treatment in comparison to other treatment, while a negative (−) Znet score represents the opposite.

A simple function was developed to evaluate the statistical significance of a two-state × two-replicate dataset, on a protein-by-protein basis, which computes an overall measurement of the system quality as the ratio of the mean of the standard deviations of the cross-state and intra-replicate populations. This is known as the ‘system to noise’ (S/N) ratio. On an individual protein level, the S/N is the ratio of the vector magnitudes (protein expression levels) across the experimental states and intra-replicate normalized values, scaled by the overall system S/N values. A simple Monte-Carlo Model was used to derived functions relating ‘false discovery rates’ (FDR) to a defined S/N cut off: all proteins with a S/N >2.8 were found to have a “false discovery rate” (FDR) of 10 % or less. This function allowed us to reliably access smaller differential expression values across experimental states, provided the variation of their corresponding intra-replicate measurements were sufficiently small.

The analysis operations, in order, were as follows: (1) The overall number of identified proteins were calculated, with the condition that each protein had to be observed in both biological replicates; (2) The overall correlation between the proteomic log2 expression values was determined; (3) Differential expression values were calculated: (a) among cross-state samples: Z = (REG-GB/REG80 glycerol), (REG-GB/REG-FFA); (REG-FFA/REG-80 glycerol); (b): among intra-replicates: R = (intra-biological replicate differences); (4) the cross-state variation was compared with the intra-replicate, variation as a “signal to noise” (S/N) quality control; (5) data sets from difference populations were transformed into final Znet scores for relative expression analyses cross-states as described by Verbeke et al. (2014). A cut-off score of ±1.65 was used to represent the outer most 10 % of the data set population defined as an asymmetry (up-regulated or down-regulated) of expression of protein relative to the expression profiles of the overall population.

## Results

### Composition of REG-80, REG-GB, and REG-FFA

The compositions of REG-80, REG-GB, and REG-FFA were provided by Renewable Energy Group (Table 1). REG-80 contained (in mass %) 80.2 % glycerol (93.7 g/L) with 0.08 % free fatty acids, 0.02 % water and 100–200 ppm methanol. REG-GB contained 45–65 % glycerol (45.2 g/L), 20–35 % free fatty acids, and up to 2500 ppm methanol. REG-FFA (free fatty acids purified from crude, biodiesel-derived glycerol) contained up to 75 % free fatty acids (9 g/L), which were predominantly C_18_ (76.60 %) and C_16_ (22.97 %) fatty acids. The concentration of free fatty acids in REG-GB was lower compared to REG-FFA (Table 1).

Our analyses indicated that medium containing 2 % (v/v) REG-80 contained 23.4 mM glycerol, while medium containing 2 % (w/v) REG-GB contained 10.4 mM glycerol, which was approximately half the glycerol concentration in REG-80 medium. No glycerol was detected in REG-FFA. REG-FFA contained 10 times more free fatty acids than REG-80 medium, and 1.5 times more free fatty acids than REG-GB medium. In addition to this, REG-80 glycerol contained 10 % potassium acetate, which was not reported in REG-FFA. Thus, the substrates used in our experiments were complex mixtures of different carbon sources, in different concentrations, with or without additional chemicals such as methanol and/or salts.

### Growth of *C. necator* H16 in REG-80, REG-GB, and REG-FFA media

**Fig. 1:**
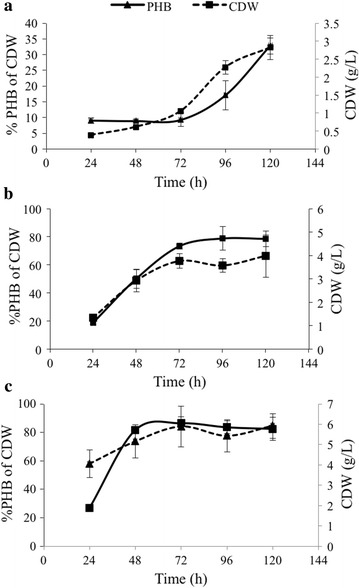
Cell dry weight (dcw) and PHB synthesis by *C. necator* H16 in RMM with **a** REG-80, **b** REG-GB, and** c** REG-FFA as substrates

All the three by-products of biodiesel production were able to support growth and PHB synthesis by *C. necator* H16 in RMM medium. However, based on the measurement of cell dry weight (cdw) at different times (Fig. 1), it is apparent that *C. necator* H16 cells were in different physiological states in media containing the different substrates at 24 and 48 h pi. In REG-80 (glycerol) cultures, total *C. necator* cdw (dried cell mass plus PHA mass), did not increase between 24 and 48 h pi (Fig. 1a). Very little total cdw (0.38 g/L cdw) and PHB accumulation (9.03 % of cdw) was observed at 24 h pi. The total cdw increased to 0.61 g/L with no further increase in PHB accumulation at 48 h pi. However, total cdw and PHB accumulation in REG-80 increased rapidly between 72 and 120 h pi, and with a maximum total cdw of 2.82 g/L and PHB accumulation of 32.85 % cdw at 120 h pi (Fig. 1a).

In contrast, the total cdw of *C. necator* H16 grown in REG-GB (glycerol bottom) and REG-FFA (free fatty acids) increased rapidly between 24 and 48 h pi. Cells grown in REG-GB produced 1.34 g/L total cdw with 18.7 % PHB accumulation at 24 h pi (Fig. 1b). The total cdw and PHB accumulation increased to up to 72 h pi with little further increase thereafter. Cells grown in REG-FFA produced 1.87 g/L total cdw and accumulated PHB to 57.9 % by 24 h pi (Fig. 1c). At 48 h pi the total cdw increased to 5.71 g/L. No further increase in cdw was observed after 48 h pi, but PHB had accumulated to 84.66 % cdw by 120 h pi.

### Consumption of glycerol, free fatty acids and nitrogen in REG-80, REG-GB, and REG-FFA cultures

**Fig. 2:**
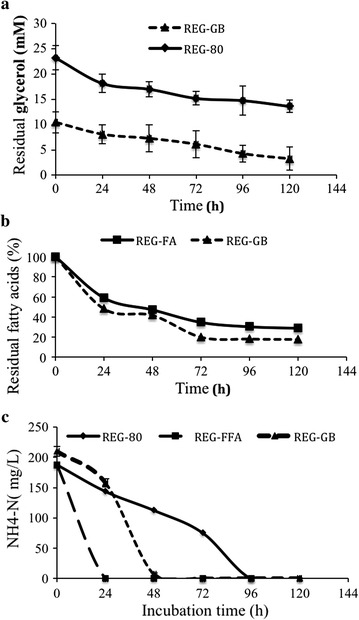
Consumption of glycerol, free fatty acids, and nitrogen by *C. necator* H16. **a** Consumption of glycerol by *C. necator* H16 in cultures containing REG-GB (glycerol bottom) and REG-80 (glycerol); **b** Consumption of fatty acids by *C. necator* H16 in cultures containing REG-GB (glycerol bottom) and REG-FFA (free fatty acids); **c** Consumption of ammonium nitrogen (NH_4_-N) by *C. necator* H16 in cultures containing REG-GB (glycerol bottom), REG-80 (glycerol), and REG-FFA (free fatty acids)

In the REG-80 cultures, only 10 mM of the glycerol substrate was consumed by *C. necator* H16 by 120 h pi, resulting in a total cell mass accumulation of 2.82 g/L (Fig. 2a). Similarly, only 7 mM of the glycerol substrate was consumed in the REG-GB cultures by 120 h pi, but the total cell mass accumulation was 3.99 g/L (Fig. 2a). Thus, the consumption of glycerol cannot account completely for the resulting cell mass production in the REG-GB culture, since the consumption of glycerol was similar for both the REG-80 and REG-GB. REG-FFA medium, which does not contain glycerol, supported the greatest cell mass accumulation (6.05 g/L).

Free fatty acids were consumed at a slightly higher rate in REG-GB media compared with REG-FFA media (Fig. 2b). At 24 h pi, 5 g/L of the initial free fatty acids in the REG-GB medium were consumed, indicating a simultaneous consumption of glycerol and free fatty acids, while 2.5 g/L of free fatty acids in REG-FFA medium were consumed in the same period (Fig. 2b). At 120 h pi, 0.90 g/L of the free fatty acids remained unutilized in the REG-GB media, while 0.98 g/L of the free fatty acids remained unutilized in the REG-FFA medium.

Consistent with the different growth rates observed (Fig. 1), the rate of ammonium nitrogen (NH_4_^+^-N) consumption and the time points at which nitrogen was depleted by *C. necator* H16 varied with the carbon source in the culture medium (Fig. 2c). NH_4_^+^-N consumption was very rapid in REG-FFA cultures and significantly slower in REG-80 cultures. Nitrogen concentrations were below detectable levels in REG-FFA cultures by 24 h pi, by 48 h pi in REG-GB cultures, and by 96 h pi in REG-80 cultures. At 24 h pi, the nitrogen concentrations remaining in REG-GB and REG-80 media were 6.9 and 112 mg/L, respectively.

### Proteomic analysis and global expression trends

Differential protein expression profiles in *C. necator* H16 cells grown in media containing REG-GB, which contains significant concentrations of both glycerol and free fatty acids were compared with the protein expression profiles from cells grown in media containing REG-FFA and REG-80 at 24 and 48 h pi. No significant differences in protein expression levels were observed in between 24 and 48 h pi for each substrate. Therefore, only the results from the 24 h pi samples are reported here (although data for the 48 h pi samples are shown in Additional file 1: Table S1). The culture conditions and time points discussed below were defined as: GB-24 (glycerol bottom) cultures at 24; FFA-24 cultures at 24; and R80-24 (glycerol) cultures at 24. Differential protein expression profiles were also compared among substrates (REG-GB, REG-FFA, and REG-80) at 24 h pi.

**Fig. 3:**
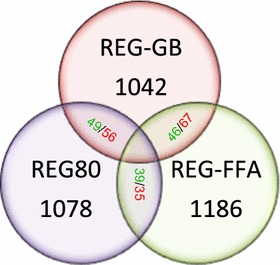
*Venn diagram* showing the number and relationship of proteins differentially expressed for the Substrate comparisons. *Circles* represent the total of proteins identified in proteome of *C. necator* H16 grown in REG glycerol bottom (REG-GB), REG free fatty acid (REG-FFA) and REG glycerol (REG-80) for 24 h. The number of proteins differentially expressed is indicated in each subset. *Green color* indicates up-regulation and *red color* indicates down-regulation. Only the 10 % of proteins showing the largest variation (Znet >± 1.65) are listed

MS/MS analyses of peptides identified between 1042 and 1233 proteins from REG-GB, REG-80, and REG-FFA grown *C. necator* H16 cells (Additional file 1: Table S1). Good correlations were observed between biological replicates and variations in within treatments (Additional file 2: Figure S1, Additional file 3: Figure S2). Protein data was further corrected for the “false discovery rate” (FDR) using the signal to noise (S/N) ratio in the cross-state versus intra-replicates, with an S/N cut-off >2.8. In this way 785, 624, and 404 differentially expressed proteins were identified in REG-GB vs REG80, REG-GB vs REG-FFA, and REG-FFA versus REG-80 at 24 h pi, respectively (Fig. 3). Relative changes in protein expression levels between substrates and sampling times observed in the outer-most 10 % (Znet ≥1.65) of the populations are given in Additional file 4: Table S2, Additional file 5: Table S3, Additional file 6: Table S4, and Additional file 7: Table S5.

Proteins were grouped according to their COG designation to identify protein level responses to changing growth phases and substrates. Cross-comparison within substrates at 24 h pi revealed 39–49 up-regulated and 35–67 down-regulated (outer 10 % proteins with Znet > ± 1.65) proteins in cells grown on the three substrates after different incubation periods (Table 2). The highest number of proteins up-regulated in GB-24 vs R-80 at 24 h pi were observed in COG C (Energy production), COG E (Amino acid and metabolism), and COG I and COG P (Lipid transport and metabolism and Inorganic ion transport and metabolism), while highly down-regulated proteins belonged to COG C (Energy production), COG O (Post translational modification, protein turn over, chaperones), and COG X (Not in COG) (Table 2). In comparison up-regulated proteins in GB-24 vs FFA-24 at 24 h pi were present in COG E, COG M (Amino acid transport and metabolism, Cell wall/membrane envelope biogenesis), and COG I (Lipid transport and metabolism). The down-regulated proteins also belonged to COG C and COG R, (Energy production and General function prediction only) and COG I (Lipid transport and metabolism), in addition to proteins of COG E (Amino acid transport and metabolism). This was expected as both the REG-GB and REG-FFA substrates were rich in fatty acids.

**Table 2:** Cross comparison of up- and down-regulated proteins in specific COG classes in *C. necator* H16 cultured with different substrates at 24 h pi

COG	Description#	GB-24 vs R80-24		GB-24 vs FFA-24		FFA-24 vs R80-24	
		Up	Down	Up	Down	Up	Down
C	Energy production	9	11	4	10	6	10
D	Cell cycle control	0	0	0	0	0	0
E	Amino acid transport and metabolism	8	6	9	8	0	0
F	Nucleotide transport and metabolism	0	0	0	0	0	0
G	Carbohydrate transport and metabolism	0	3	1	2	2	1
H	Coenzyme transport and metabolism	4	1	0	0	0	0
I	Lipid transport and metabolism	6	2	5	9	8	1
J	Translation, ribosomal structure and biogenesis	2	1	2	1	0	0
K	Transcription	1	2	2	1	0	2
L	Replication, recombination and repair	0	0	2	0	0	0
M	Cell wall/membrane envelope biogenesis	0	0	7	1	4	0
N	Cell motility	1	2	1	2	0	0
O	Post translational modification, protein turn over, chaperone	0	10	1	3	1	8
P	Inorganic ion transport and metabolism	6	1	0	0	3	0
Q	Secondary metabolites biosynthesis, transport and catabolism	1	0	2	4	0	0
R	General function prediction only	2	3	2	10	5	3
S	Function unknown	4	5	0	0	2	1
T	Signal transduction mechanism	0	0	1	3	0	0
U	Intracellular trafficking, secretion and vesicular transport	1	2	0	5	0	3
V	Defense mechanism	0	0	2	1	0	2
X	Not in COG	4	7	5	7	8	4
	Total	49	56	46	67	39	35

### PHB synthesis proteins

Three enzymes are involved in PHB synthesis in *C. necator* H16: PhbA, PhbB, and PhbC. PhbA, (encoded by *phb*A) is a ketothiolase, which combines 2 acetyl–CoA to form acetoacetyl-CoA. PhbB, encoded by *phb*B, is an Acetoacetyl-CoA reductase, which reduces acetoacetyl-CoA to 3-hydroxybutynyl-CoA. Finally, PhbC, encoded by two genes, is a heterodimeric polyhydroxybutyrate synthase, which polymerizes the 3-hydroxybutynyl-CoA monomers to PHB (Table 4). These genes are encoded by the PHB operon, *phbCAB* (H16_A1437; H16_A1438; H16_A1439, and H16_A1440) and their role in PHB synthesis was confirmed earlier by cloning and expression in *E. coli* (Peoples and Sinskey 1989; Schubert et al. 1988). All three PHB-biosynthetic enzymes (PhbA, PhbB, and PhbC) are synthesized constitutively and the expression of the PHB-biosynthetic genes is regulated at both the transcriptional or translational levels (Haywood et al. 1988, 1989; Schubert et al. 1991). PHB accumulation is regulated by the kinetic parameters of the three enzymes, which are influenced by intracellular concentrations of substrates and cofactors (Peoples and Sinskey 1989).

Each of the gene products encoded by the four genes of the PHB operon, plus two PHB depolymerase gene products and three Phasin proteins were expressed by *C. necator* H16 in REG-GB, REG-80, and REG-FFA cultures (Table 4). Although none of the PHB synthesis enzymes showed significant changes in their expression levels in REG-GB or REG-FFA cultures when compared to REG-80 cultures, one Phasin protein, PhbP1 (H16_A1381), was highly up-regulated in REG-GB and REG-FFA cultures (compared with REG-80 cultures). Another Phasin Protein PhbP4 (H16_B2021) was down-regulated in REG-GB cultures when compared with REG-80 cultures (Table 3).

**Table 3:** Differential protein expression levels (Pnet) associated with poly-(3-hydroxybutyrate) synthesis and other accessory proteins of PHB granules in *C. necator* H16 cultured with different substrates

Locus tag	Protein	GB-24/R80-24	GB-24/FFA-24	FFA-24/R80-24
H16_A1437	Poly(3-hydroxybutyrate) polymerase PhaC	0.35	−0.41	−0.14
H16_A1438	Acetyl-CoA acetyltransferase PhaA	−0.07	0.08	−0.15
H16_A1439	Acetoacetyl-CoA reductase PhaB	−0.21	−0.01	−0.04
H16_A1440	transcriptional regulator of phasin expression PhaR	0.26	−0.29	−0.35
H16_A1381	Phasin (PHA-granule associated protein) PhaP1	2.96*	0.27	*2.68*
PHG202	Phasin (PHA-granule associated protein) PhaP2	ND	ND	ND
H16_A2172	Phasin (PHA-granule associated protein) PhaP3	ND	ND	ND
H16_B2021	Phasin (PHA-granule associated protein) PhaP4	*−1.84*	−0.58	0.88
H16_B1934	Phasin (PHA-granule associated protein) PhaP5	−0.06	ND	−0.51
H16_A2862	Intracellular poly(3-hydroxybutyrate) depolymerase	ND	0.34	ND
H16_A1150	Poly(3-hydroxybutyrate) depolymerase	0.27	−0.02	0.41
H16_B0339	Poly(3-hydroxybutyrate) depolymerase	ND	ND	ND
H16_B1014	Poly(3-hydroxybutyrate) depolymerase	ND	ND	ND
H16_B2073	Poly(3-hydroxybutyrate) depolymerase	ND	ND	ND
H16_B2401	Poly(3-hydroxybutyrate) depolymerase	ND	ND	ND
H16_A0141	Membrane associated protein PhaM	−1.39	−0.62	−0.49

Up- or down-regulation is limited to outer 10 % proteins with a relative Z-score expression ratio (Znet) outside (> ±1.65) from the population mean; *italic* numbers indicate significant up- or down-regulation of protein expression

### Glycerol metabolism proteins

**Fig. 4:**
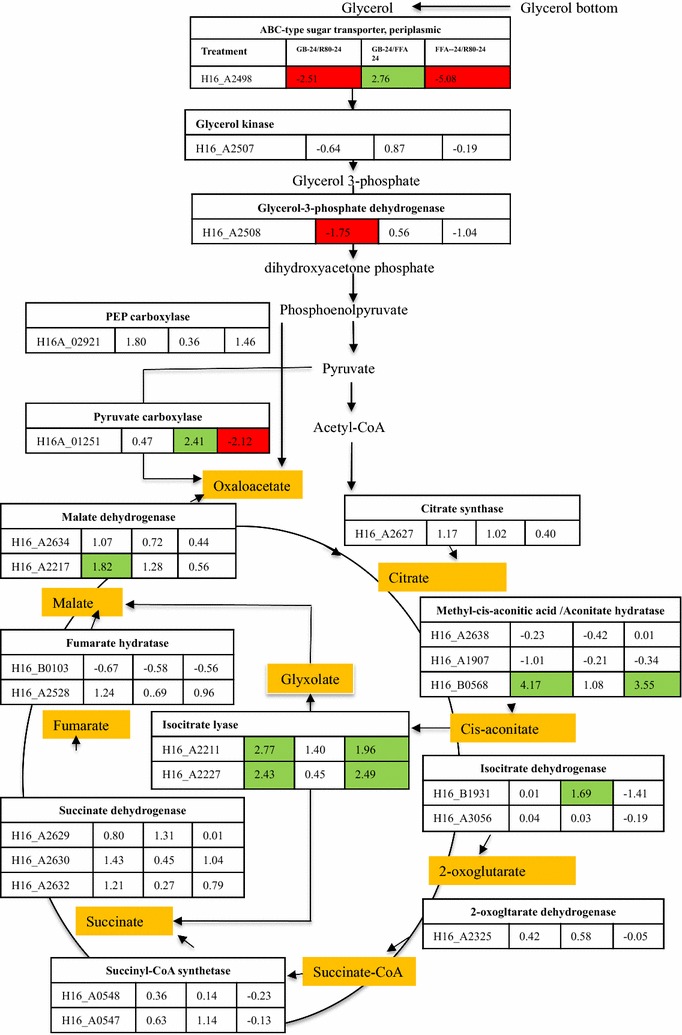
Differential expression level of proteins of glycerol metabolism pathway, the TCA cycle, and glyoxylate shunt pathway in *C. necator* H16 grown in REG-GB, REG-FFA and REG-80 at 24 h pi. Expression levels are presented as normalized relative Z-score expression ratios (Znet >1.65). The *green colored numbers* indicate up-regulation of protein expression; *red colored numbers* indicate down-regulation of protein expression. *White color* no significant change. GB-24, REG-GB (glycerol bottom) at 24 h pi; R80-24, REG-80 (glycerol) at 24 h pi; FFA-24, REG-FFA (free fatty acids) at 24 h pi

Glycerol transport across the cytoplasmic membrane occurs through facilitated diffusion mediated by the glycerol uptake facilitator protein GlpF (Sweet et al. 1990; Darbon et al. 1999). This protein (H16_A3690) was not detected in proteomes of *C. necator* H16 grown on any of the three substrates used in this work. Another ABC-type transporter gene, identified as *glp*V (H16_A2498), encodes another glycerol transport system substrate-binding protein that was down-regulated in REG-GB and REG-FFA cultures compared to the REG-80, but up-regulated compared to REG-FFA cultures (Fig. 4). It appears that this protein is inducible by glycerol, which was present in REG-GB and REG-80, but not in REG-FFA.

Two sets of genes (H16_A2507-H16_A2508 and H16_B1199-H16_B11980) that are involved in glycerol uptake and metabolism were identified in *C. necator* H16 (Darbon et al. 1999). These genes encode a putative glycerol kinase (GlpK) and a putative FAD-dependent glycerol 3-phosphate dehydrogenase (GlpD). Both the genes were required for glycerol metabolism and in the absence of GlpD glycerol-3-phosphate accumulated in the cell. The glycerol kinase (H16_A2507 and H16_B1199) and glycerol-3-phosphate dehydrogenase (H16_A2508 and H16_A0336) are involved in the phosphorylation of intracellular glycerol to glycerol 3-phosphate, and the subsequent conversion to dihydroxyacetone phosphate. In REG-GB, REG-FFA, and REG-80 at 24 h pi grown cells, glycerol kinase (H16_A2507) was not changed significantly and the other glycerol kinase (H16_AB1199) was not detected in the proteomes. Glycerol-3-phosphate dehydrogenase (H16_A2508) was also down-regulated in REG-GB cultures compared with REG-80 cultures. Glycerol-3-phosphate is converted into dihydroxyacetone phosphate, which is introduced into gluconeogenesis or catabolized through the ED pathway via pyruvate to acetyl-CoA, the precursor for the tricarboxylic acid cycle and for PHB biosynthesis. Our proteomic data, however, showed that glycerol kinase (H16_A2507) and glycerol-3-phosphate dehydrogenase (H16_A2508) were expressed in *C. necator* H16 proteomes. However, higher expression of glycerol transporter was not associated with higher expression of glycerol utilizing proteins.

### β-oxidation proteins in *C. necator* H16

*Cupriavidus necator* H16 can grow and produce PHB from vegetable oils (triacylglycerides). Use of vegetable oil triacylglycerides as a carbon source, however, requires cleavage of the fatty acid chains from glycerol before they may be metabolized via the β-oxidation pathway. Two lipase genes (H16_A1322 and H16_A3742) were identified previously in *C. necator* H16 transcriptomes from cells grown on palm oil (Brigham et al. 2010). However, neither of these proteins was present in the *C. necator* proteomes derived from cells grown on the three substrates tested, most likely because these substrates contained free fatty acids instead of triglycerides.

**Fig. 5:**
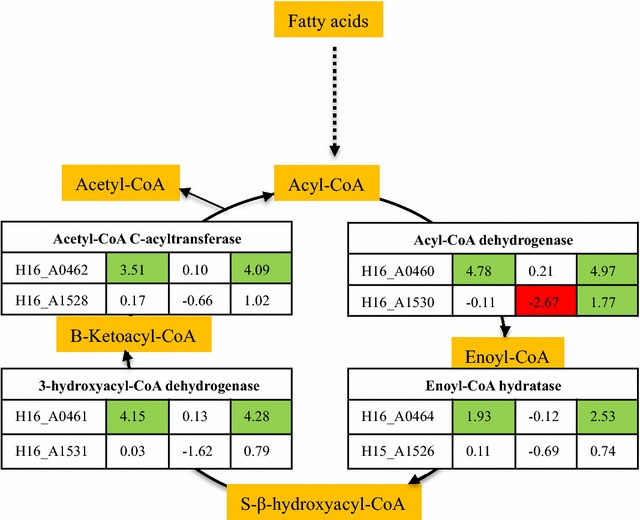
Differential protein expression levels in the fatty acid oxidation pathway in *C. necator* H16 grown with different substrates at 24 h pi. Expression levels are presented as normalized relative Z-score expression ratios (Znet >1.65). The *green colored numbers* indicate up-regulation of protein expression; *red colored numbers* indicate down-regulation and *white color* no significant change of protein expression. GB-24, REG-GB (glycerol bottom) at 24 h pi; R80-24, REG-80 (glycerol) at 24 h pi; FFA, REG-FFA (free fatty acids) at 24 h pi

The β-oxidation pathway genes are present in two operons (H16_A0459-H16_A0464 and H16_A1526-H16_A1531) encoded by the *C. necator* H16 genome, and have been detected in previous *C. necator* proteomes (Pohlmann et al. 2006). In the current study, all the β-oxidation pathway gene products—the acyl-CoA dehydrogenases (FadE, H16_A0460 and H16_A1530), the 2-enoyl-CoA hydratases (FadB, H16_A0464 and H16_A1526), the 3-hydroxyacyl-CoA dehydrogenases (FadB, H16_A0461 and H16_A1531), and the 3-ketoacyl-CoA thiolases (FadB, H16_A0462 and H16_A1528) were detected in *C. necator* H16 cells cultured with REG-GB, REG-80, and REG-FFA (Fig. 5).

Between the two β-oxidation operons, proteins of one operon (H16_A0459-H16_A0464) were highly up-regulated in REG-GB and REG-FFA cultures in comparison to REG-80 cultures at 24 h pi. From the other operon (H16_A1526-H16_A1531), only acyl-CoA dehydrogenase (H16_A1530) was up-regulated in REG-FFA cultures compared with REG-80 cultures (Fig. 4). Recently, Insomphun et al. (2014) identified another operon associated with β-oxidation (H16_B0721-H16_B0724). In this operon, FadB (enoyl-CoA hydratase H16_B0724) was present with two acyl-CoA dehydrogenases (H16_B0721-H16_B0722). No protein from this operon was detected in the current *C. necator* H16 proteomes. The expression of β-oxidation pathway proteins (H16_A0459-H16_A0464) in REG-GB was up-regulated at 48 h pi in comparison to REG-80 cultures, but no changes were observed in REG-FFA cultures in comparison to REG-GB. The proteins of the other β-oxidation pathway (H16_A1526-H16_A1531) were not changed significantly in REG-GB and were down-regulated in REG-FFA cultures (Fig. 5).

### Expression of hydrogenase proteins

The facultative chemolithoautotrophic proteobacterium *C. necator* H16 is able to use hydrogen as the sole energy source in aerobic environments. *Cupriavidus necator* H16 is known to have three distinct [NiFe]-hydrogenases which serve unique physiological roles: a membrane-bound hydrogenase (MBH) coupled to the respiratory chain; a cytoplasmic, soluble hydrogenase (SH) able to generate reducing equivalents by reducing NAD^+^ at the expense of H_2_; and a regulatory hydrogenase (RH), which acts in a signal transduction cascade to control hydrogenase gene transcription. The genes for these hydrogenases are present on plasmid pHG1 (Schwartz et al. 2003).

Both autotrophic growth and heterotrophic growth are known to induce hydrogenases. Ten (10) hydrogenase-related proteins were identified in the current *C. necator* H16 proteomes, including HoxG, HypD, HoxA, HoxC, HoxF, HypB1, HypB2, HoxI, and HoxH (Table 4). The *hox* regulon contains the structural and auxiliary genes for hydrogenase assembly. Expression levels of all 10 hydrogenase-related proteins were very low in REG-GB and REG-FFA cultures, possibly because the preferential carbon source fatty acids repressed the expression of these genes. In contrast, more than 80 % of the usable substrate in REG-80 was glycerol, with only trace amounts of fatty acids. Judger et al. (2015) observed the derepression of hydrogenase proteins during diauxic growth after fructose depletion. The low concentrations of free fatty acids in REG-80 culture could have contributed to derepression of the hydrogenase genes.

**Table 4:** Differential expression of proteins associated with hydrogenase activity in *C.necator* H16 grown in REG glycerol bottom in comparison to REG-FFA and REG80 glycerol cultures after 24 h pi

Accession #	Protein	Pnet scores		
		GB-24/R80-24	FFA-24/R80-24	GB-24/FFA-24
PHG002	Membrane bound (NiFe) hydrogenase large unit	*−4.04*	*−4.96*	ND
PHG019	Hydrogenase transcriptional regulatory protein, HoxA	*−1.53*	−1.79	ND
PHG016	[NiFe] hydrogenase metallocenter assembly protein HypD1	*−1.79*	*−2.13*	ND
PHG021	Regulatory [NiFe] hydrogenase large subunit	*−1.98*	*−3.13*	−0.84
PHG088	NAD-reducing hydrogenase diaphorase moiety large	*−4.55*	*−5.59*	ND
PHG095	[NiFe] hydrogenase nickel incorporation-associated protein HypB2	*−2.06*	*−2.53*	ND
PHG013	[NiFe] hydrogenase nickel incorporation-associated protein HypB1	*−3.23*	*−4.99*	−0.36
PHG091	NAD-reducing hydrogenase hydrogenase moiety	*−4.43*	*−5.56*	ND
PHG_00093	HoxI	*−6.79*	*−5.39*	−1.07

Up- or down-regulation is limited to outer 10 % proteins with a relative Z-score expression ratio (Pnet) outside (> ±1.65) from the population mean; *italic* numbers indicate significant up or down regulation of protein expression

## Discussion

The objective of this study was to correlate differences in the physiological states of *C. necator* H16 grown with different biodiesel waste by-products with changes in protein expression level. The three substrates used represent complex mixtures of carbon sources, which effected microbial metabolism in different ways. REG-80 consisted of 80 % glycerol, with negligible amounts of free fatty acids (0.08 %). REG-GB contained up to 65 % glycerol and 35 % free fatty acids, while REG-FFA contained up to 75 % FFA and no glycerol. All the three substrates supported growth and PHB production by *C. necator*, but substrate-specific effects were observed for cell mass production, PHB accumulation, and protein expression levels of several key metabolic pathways, including glycerol metabolism, the fatty acid β-oxidation pathway, the Glyoxylate Shunt, and hydrogen (H_2_) synthesis pathways.

### Expression of proteins related to PHB synthesis and accumulation

Raberg et al. (2011) compared the proteomes of *R. eutropha* H16 and a PHB^−^ mutant and concluded that PHB synthesis proteins were not expressed at higher levels in PHB synthesizing strains compared with the non-PHB-synthesizing strain. While our results were consistent with these findings with respect to PhbA, PhbB, and PhbC, we observed significant up-regulation of the Phasin P1 protein, and down-regulation of the Phasin P4 protein in REG-GB and REG-FFA cultures. Phasins are small proteins associated with inclusion bodies in bacterial cells. Most are granule-associated proteins involved in accumulation of polyhydroxyalkanoate polymers, like PHB. Of the seven Phasin proteins (PhaP1-PhaP7) encoded in the *C. necator* genome, only two, PhaP1 and PhaP4 (H16_A1381 and H16_B2021), were detected in our *C. necator* H16 proteomes. PhaP1 was up-regulated in REG-GB and REG-FFA in comparison to REG-80 cultures. This may be expected, as both of these cultures were actively accumulating PHB at the sampling time (t = 24 h pi), which correlated to exponential growth in the REG-GB and REG-FFA cultures. In contrast, REG-80 cultures were still in lag-phase at 24 h pi. Potter and Steinbuchel (2005) described the PhaP3 as a homologue of PhaP1, which is normally present in low amount in cell producing PHB and have different role in the cell (Pfeiffer and Jendrossek 2012). In a mutant where *pha*P1 was deleted, expression of *pha*P3 increased significantly. In our proteomes, PhaP3 was not detected, possibly due to very low levels of expression or higher levels of expression of PhaP1.

### Expression of proteins related to regulation of nitrogen metabolism

PHB accumulation starts when nitrogen concentrations are depleted from the medium (Ramsay et al. 1991; Sharma et al. 2012). However, as noted above, the rate of nitrogen consumption and the time point at which nitrogen was depleted by *C. necator* H16 varied with the carbon source in the culture medium. Ammonium nitrogen (NH_4_^+^-N) concentrations were below detectable levels in REG-FFA cultures by 96 h pi in REG-80 cultures, by 48 h pi in REG-GB cultures, and before 24 h pi in REG-FFA cultures. Available nitrogen in the media affects both cell growth and PHB accumulation, and high(er) nitrogen concentrations in cultures have been shown to increase the growth and PHB production of *C. necator* grown on oil (Wang and Yu 2001; Wang et al. 2007). *Cupriavidus necator* H16 produced PHB from vegetable oils in Tryptone Soya Broth containing 4.4 g/L nitrogen (Verlinden et al. 2011; Obruca et al. 2010). Likewise, Kahar et al. (2004) used nitrogen excess medium (4 g/L) to produce PHB in fed batch cultures using soybean oil.

A nitrogen regulatory protein P-II (*gln*K) that is sensitive to nitrogen concentrations, encoded at locus tag H16_A0320 of the *C. necator* H16 genome was highly up-regulated. PII proteins act as sensors of cellular nitrogen status and are regulated by covalent modifications, like uridylation. This protein was identified as an energy and nitrogen sensor in bacteria (Commichau et al. 2006; Javelle et al. 2004). The expression of the PII protein was previously reported to represses transcription of the PHB synthesis operon (Schwartz et al. 2009). At 24 h pi, this regulatory protein was expressed at very high levels in REG-GB (Znet = 3.31) and REG-FFA (Znet = 3.71) cultures relative to REG-80 cultures. However, expression levels of the PHB synthesis proteins in *C. necator* remained unchanged (Additional file 5: Table S3, Additional file 6: Table S4, and Additional file 7: Table S5).

### Expression of proteins related to glycerol metabolism

Unlike *C. necator* JMP134 and *C. necator* DSM545, *C. necator* H16 is not an efficient glycerol user (Lopar et al. 2013; Mothes et al. 2007). Glycerol is not a preferred carbon source for growth of *C. necator* H16, and when cultured in glycerol containing biodiesel by-products, *C. necator* H16 cells are always under stress. Reactive Oxygen Species (ROS) may be produced when *C. necator* H16 is grown in glycerol and in response, proteins like catalases, peroxidases, and hydrogenases, are induced (Additional file 7: Table S5).

Glycerol-3-phosphate dehydrogenase was expressed a high levels in REG-80 cultures compared with REG-GB and REG-FFA cultures, and as expected, expression of glycerol metabolizing proteins was lowest in REG-FFA cultures, due to the absence of glycerol in this substrate. Low expression of glycerol kinase has been correlated with limited growth of *C. necator* H16 on glycerol. In contrast, recombinants that expressed *glp*F and *glp*K from *E. coli* grow at a faster rate (Fukui et al. 2014). *Cupriavidus necator* H16 has also been shown to use an alternative glycerol uptake system like PEP-PTS for glycerol transport (Kaddor and Steinbuchel 2011). In REG-GB cultures, a serine kinase of the Hpr protein (H16_A0383) and phosphotransferase system (H16_A0384), related to alternate PEP-PTS, were identified, but not expressed at significantly greater levels at 48 h pi with any of the substrates.

### Expression of proteins related to the β-oxidation pathway

As expected, accumulation of PHB was higher in REG-FFA cultures than in REG-GB and REG-80 cultures at 24 pi. Most of the triglycerides were hydrolyzed in REG-GB and REG-FFA, therefore no induction of lipases (H16_A1322, H16_A1323) was observed. The β-oxidation pathway provided the precursor for PHB synthesis when cultivated on REG-GB and REG-FFA. It appears that activities of β-oxidation enzymes were maximum and no further up-regulation was required.

Of the two operons involved in β-oxidation, the enzymes of operon H16_A0459- H16_A0464 were highly up-regulated in REG-GB cultures, while the other operon (H16_A1526-H16_A1531) proteins were not expressed at same level in late exponential phase (t = 48 h pi). Mutants with deletions in H16_A0459-H16_A464 or H16_A1526-H16_A1531 did not show any decrease in growth or PHB accumulation from oils and both the operon complement each other (Brigham et al. 2010). Up-regulation of proteins of the H16_0459-H16_464 operon were observed, with no change in protein expression levels of the H16_A1526- H16_A1531operon. The reason for this selective up-regulation of β-oxidation proteins is not known. The presence of fatty acids in the REG-GB and REG-FFA media could act as an inducer of the β-oxidation pathway, resulting in very high levels of expression of these proteins.

The β-oxidation pathway in *C. necator* H16 is similar to the well-studied pathway of *Escherichia coli* with two *fad* operons that are induced by fatty acids containing 12 or more carbon atoms (Schubert et al. 1988). The genome sequence of *C. necator* H16 encodes a number of genes responsible for the degradation of fatty acids in this organism, and different sets of genes are expressed under different growth conditions. For example, expression of β-oxidation proteins encoded by operon H16_A0459-H16_A0464 was higher in REG-GB as well as REG-FFA grown cultures but not of other operon H16_A1526-H16_A1531 proteins. Proteins of fatty acid β-oxidation pathway were highly expressed in REG-FFA and REG-GB cultures compared to REG-80 cultures.

Both the substrates were rich in fatty acids and presence of fatty acids is known to induce β-oxidation proteins. Acetyl CoA produced by β-oxidation is converted to oxaloacetate by Glyoxylate Shunt. Wang et al. (2003) and Brigham et al. (2010) observed decreased growth of *C. necator* on oil when aceB gene (malate synthase) was deleted. We did not find any change in AceB protein expression in any substarates. However, in our proteomes two proteins IclA (H16_A2211), IclB (H16_A2227) designated as isocitate lyase were highly expressed in RG-GB and RG-FFA media. In addition to this, another protein, AcnB (aconitate hydratase) was also highly upregulated in GB-RG and RG-FFA. These results were consistent with Brigham et al. (2010), who showed (using microarray studies) that transcription of genes in the Glyoxylate Shunt pathway was up-regulated when culture was grown on trioleate. In our proteomes, however, different proteins of Glyoxylate cycle, which were not reported earlier, were highly up-regulated. The Glyoxylate Shunt pathway is known to play a major role in utilization of fatty acids, which are metabolized to acetyl-CoA, a key intermediate for synthesis of many cellular components (Brigham et al. 2010). REG-FFA and REG-GB medium had higher concentrations of free fatty acids than REG-80 medium and therefore β-oxidation was more prevalent in REG-FFAand RG-GB grown cultures.

In conclusion, one-dimensional liquid chromatography followed by mass spectroscopy (1D/LC/MS/MS) analyses of *C. necator* H16 cultured with biodiesel production by-products identified proteins of key metabolic pathways. Cell growth, PHB accumulation, and gene product expression patterns were similar in *C. necator* H16 cultures grown on REG-GB and REG-FFA, but these were very different in cultured grown on REG-80. Low levels of biomass production, PHB accumulation, and up-regulated expression of hydrogenase in REG-80 cultures confirmed that glycerol is a sub-optimal carbon source for *C. necator* H16. In contrast, *C. necator* H16 cells grew faster, achieved greater cell densities, and accumulated PHB to a much greater percentage of the cell dry weight in REG-FFA media, which contained biodiesel-derived free fatty acids. Thus, REG-FFA appears to be an effective, low cost substrate for PHB production.

PHB synthesis enzymes (PhbA, PhbB, and PhbC) and two PHB depolymerase gene products were expressed in *C. necator* H16 cultures containing glycerol (REG-80), glycerol and free fatty acids (REG-GB), and free fatty acids (REG-FFA), but no significant changes in expression levels were observed. However, expression levels of the Phasin P1 protein were significantly increased during active PHB accumulation in REG-GB and REG-FFA cultures cultured compared with REG-80 cultures. In contrast, expression levels of the Phasin P4 protein were significantly decreased in REG-GB cultured compared with REG-80 cultures.

## Additional files

10.1186/s13568-016-0206-z P-scores of proteomic runs of *C. necator* H16 grown with different substrates.
10.1186/s13568-016-0206-z Intra- and inter-replication variation among different replications and treatment of C.necator H16. Comparison is presented for C. necator H16 grown with: REG-FFA vs REG-80 at 24h pi. Log2 expression values of replication 2 (x-axis) are plotted against replication 1 (y-axis). Blue dots are intra replication variations and orange dots are inter- replicate variations.
10.1186/s13568-016-0206-z Linear regression analysis of the log2 expression values for all observed proteins between biological replicates grown with REG-FFA and REG-80 at 24h pi. Log2 expression values observed for biological replicate 1 are plotted on the x-axis, while the corresponding values for biological replicate 2 are plotted on the y-axis.
10.1186/s13568-016-0206-z Differential protein expression in *C. necator* H16 grown with REG-GB in comparison to REG-80 at 24 h pi.
10.1186/s13568-016-0206-z Differential protein expression in *C. necator* H16 grown with REG-GB in comparison to REG-FFA after 24h pi.
10.1186/s13568-016-0206-z Differential protein expression in *C. necator* H16 grown with REG-FFA in comparison to REG-80 after 24h pi.
10.1186/s13568-016-0206-z Highly expressed proteins (up- or down-regulated) in *C. necator* H16 grown with REG-GB in comparison to REG-FFA and REG-80 at 24 h pi.
