# Supplementary material for: Global changes in the proteome of Cupriavidus necator H16 during poly-(3-hydroxybutyrate) synthesis from various biodiesel by-product substrates
Source: AMB Express. 2016 May 17;6:36. doi: 10.1186/s13568-016-0206-z (PMC4870535; doi:10.1186/s13568-016-0206-z)
Supplement: Supplementary file 1 — 10.1186/s13568-016-0206-z P-scores of proteomic runs of C. necator H16 grown with different substrates. [file 13568_2016_206_MOESM1_ESM.docx]

**Supplementary Tables**

**Table S1.** P-scores of proteomic runs of *C. necator* H16 grown with different substrates.

**Supplementary Table 1.** P-scores of proteomic runs of *C. necator* H16 grown with different substrates.

| Experimental Condition | MS/MS Spectra  Collected | Total # Peptides  Detected | # Non- redundant Peptides  Detected | # of Proteins at: EV < -1 | # of Proteins at: EV < -3 | # of Proteins at: EV < -10 |
| --- | --- | --- | --- | --- | --- | --- |
| GB-24-1# | 25858 | 16155 | 7016 | 1439 | 1338 | 1042 |
| GB-24-2* | 23741 | 15550 | 1887 | 878 | 725 | 437 |
| FFA-24-1 | 32896 | 18752 | 7479 | 1674 | 1540 | 1178 |
| FFA-24-2 | 34826 | 19838 | 7046 | 1664 | 1552 | 1184 |
| REG80-24-1 | 20339 | 13384 | 6358 | 1518 | 1404 | 1087 |
| REG80-24-2 | 21199 | 13381 | 6439 | 1482 | 1366 | 1070 |

# 24-1 Sampled at 24h pi, replication 1, 24-2 Sampled at 24h pi , replication 1

* GB-24-2 replication low protein number and not included in calculation
