# Supplementary material for: Global changes in the proteome of Cupriavidus necator H16 during poly-(3-hydroxybutyrate) synthesis from various biodiesel by-product substrates
Source: AMB Express. 2016 May 17;6:36. doi: 10.1186/s13568-016-0206-z (PMC4870535; doi:10.1186/s13568-016-0206-z)
Supplement: Supplementary file 2 — 10.1186/s13568-016-0206-z Intra- and inter-replication variation among different replications and treatment of C.necator H16. Comparison is presented for C. necator H16 grown with: REG-FFA vs REG-80 at 24h pi. Log2 expression values of replication 2 (x-axis) are plotted against replication 1 (y-axis). Blue dots are intra replication variations and orange dots are inter- replicate variations. [file 13568_2016_206_MOESM2_ESM.docx]

##### **Supporting information**

**Supplementary Figures**

###### **Figure S1.** Intra- and inter-replication variation among different replications and treatment of C.necator H16. Comparison is presented for C. necator H16 grown with: REG-FFA vs REG-80 at 24h pi. Log2 expression values of replication 2 (x-axis) are plotted against replication 1 (y-axis). Blue dots are intra replication variations and orange dots are inter- replicate variations.
