# Supplementary material for: Global changes in the proteome of Cupriavidus necator H16 during poly-(3-hydroxybutyrate) synthesis from various biodiesel by-product substrates
Source: AMB Express. 2016 May 17;6:36. doi: 10.1186/s13568-016-0206-z (PMC4870535; doi:10.1186/s13568-016-0206-z)
Supplement: Supplementary file 3 — 10.1186/s13568-016-0206-z Linear regression analysis of the log2 expression values for all observed proteins between biological replicates grown with REG-FFA and REG-80 at 24h pi. Log2 expression values observed for biological replicate 1 are plotted on the x-axis, while the corresponding values for biological replicate 2 are plotted on the y-axis. [file 13568_2016_206_MOESM3_ESM.docx]

**Figure S2.** Linear regression analysis of the log2 expression values for all observed proteins between biological replicates grown with REG-FFA and REG-80 at 24h pi. Log2 expression values observed for biological replicate 1 are plotted on the x-axis, while the corresponding values for biological replicate 2 are plotted on the y-axis.
