# Supplementary material for: Global changes in the proteome of Cupriavidus necator H16 during poly-(3-hydroxybutyrate) synthesis from various biodiesel by-product substrates
Source: AMB Express. 2016 May 17;6:36. doi: 10.1186/s13568-016-0206-z (PMC4870535; doi:10.1186/s13568-016-0206-z)
Supplement: Supplementary file 4 — 10.1186/s13568-016-0206-z Differential protein expression in C. necator H16 grown with REG-GB in comparison to REG-80 at 24 h pi. [file 13568_2016_206_MOESM4_ESM.docx]

**Supplementary Tables**

**Table S2.** Differential protein expression in *C. necator* H16 grown with REG-GB in comparison to REG-80 at 24 h pi.

**Supplementary Table S2.** Highly expressed proteins (up- or down-regulated) in *C. necator* H16 grown on REG-GB in comparison to REG-FFA and REG-80 at 24 h pi

| **Locus tag** | **Protein** | **GB-24 / R80-24*** | **FFA-24 / R80-24** | **GB-24 / FFA-24** |
| --- | --- | --- | --- | --- |
| H16_A0462 | Acetyl-CoA C-acyltransferase | **3.51** | **4.09** | 0.1 |
| H16_A0460 | Acyl-CoA dehydrogenase | **4.78** | **4.97** | 0.21 |
| H16_A0461 | two domain protein: 3-hydroxyacyl-CoA dehydrogenase | **4.15** | **4.28** | 0.13 |
| H16_A0464 | Enoyl-CoA hydratase/carnithine racemase | **1.93** | **2.53** | -0.12 |
| H16_A2119 | Acyl-CoA-binding protein | **2.92** | **3.77** | -1.03 |
| H16_A1332 | Succinyl-CoA:3-ketoacid-coenzyme A transferase | **2.02** | **0.71** | 1.41 |
| H16_A3514 | Long-chain-fatty-acid-CoA ligase | **1.67** | ND | **2.29** |
| H16_B0834 | Acetate-CoA ligase | 1.38 | -0.72 | **3.03** |
| H16_A2211 | Isocitrate lyase | **2.77** | **1.96** | 1.40 |
| H16_A2227 | Isocitrate lyase | **2.43** | **2.49** | 0.45 |
| H16_A1075 | ABC-type transporter, periplasmic component: | **3.51** | **5.09** | -0.89 |
| H16_A1035 | Acetolactate synthase III (valine sensitive) | **2.25** | **2.28** | **1.96** |
| H16_A0320 | Nitrogen regulatory protein PII glnK | **3.31** | **3.71** | 0.06 |
| H16_B1945 | Glutamate dehydrogenase [NAD(P)+] | **3.08** | 0.66 | **3.07** |
| H16_A3030 | ABC-type transporter, periplasmic component: | **2.65** | **2.94** | 0.84 |
| H16_B1078 | Extra-cytoplasmic solute receptor (Urea transport) | 1.66 | ND | **2.30** |
| H16B_01596 | hypothetical membrane associated protein | 2.53 | 0.88 | **2.21** |
| H16_B2566 | Glucose-6-phosphate 1-dehydrogenase | **-1.37** | -0.02 | -1.36 |
| H16_A2508 | Glycerol-3-phosphate dehydrogenase | **-1.75** | -1.04 | ND |
| H16_A1178 | Phosphogluconate dehydratase | **-1.54** | 0.08 | ND |
| H16_A2498 | ABC-type sugar transporter, periplasmic glycerol transport | **-2.51** | **-5.08** | **2.76** |
| PHG002 | Membrane bound (NiFe) hydrogenase large unit | **-3.8** | **-4.96** | ND |
| PHG019 | Hydrogenase transcriptional regulatory protein, HoxA | **-1.42** | -1.57 | ND |
| PHG016 | [NiFe] hydrogenase metallocenter assembly protein HypD | **-1.72** | **-2.13** | ND |
| PHG021 | Regulatory [NiFe] hydrogenase large subunit | **-1.92** | **-3.13** | -0.84 |
| PHG088 | NAD-reducing hydrogenase diaphorase moiety large | **-4.19** | **-5.59** | ND |
| PHG095 | [NiFe] hydrogenase nickel incorporation-associated protein HypB2 | **-2.33** | **-2.53** | ND |
| PHG013 | [NiFe] hydrogenase nickel incorporation-associated protein HypB1 | **-3.02** | **-4.98** | -0.78 |
| PHG091 | NAD-reducing hydrogenase hydrogenase moiety | **-4.55** | **-5.55** | ND |
| H16_A3102 | Glutathione peroxidase | **-2.50** | **-1.73** | -040 |
| H16_B2041 | ABC-type transporter, branched chain amino acids | **-2.07** | -0.39 | **-3.06** |
| H16_A0252 | Glutathione S-transferase | **-2.09** | -1.12 | -1.49 |
| H16_B2428 | ATP-dependent protease Clp, ATPase subunit | **-2.71** | **-2.96** | ND |
| H16_B2239 | Peroxiredoxin | **-2.92** | **-2.01** | -1.00 |
| H16_A2213 | Non-heme haloperoxidase | **-1.31** | **-5.43** | **4.66** |
| H16_A2777 | Catalase (peroxidase I) | **-2.69** | ND | ND |
| H16_A1910 | Glutaminase-asparaginase (amidohydrolase) | **-3.74** | -1.46 | **-2.46** |
| H16_A0982 | flp pilus assembly protein secretin CpaC | **-3.36** | -0.61 | **5.73** |
| H16_A1381 | Phasin (PHA-granule associated protein) | **2.96** | 3.01 | 0.27 |
| H16A_03701 | putative peptidoglycan binding domain | **-2.36** | -1.42 | 0.39 |
| H16A_00011 | Hypothetical protein | **-2.63** | -0.59 | **-2.19** |
| H16B_00105 | Ferredoxin | **-3.55** | -1.17 | **-2.53** |
| H16A_00977 | hypothetical protein | **-3.83** | **-2.10** | **-2.6** |
| H16_A3030 | ABC-type transporter, periplasmic component: | **2.65** | **2.94** | 0.06 |
| H16_A2498 | ABC-type transporter, periplasmic component | **2.51** | **-4.11** | **2.76** |
| H16_A2232 | ABC-type transporter, periplasmic component: | **2.30** | 0.95 | **1.92** |
| H16_A0359 | ABC-type transporter, periplasmic component: | **2.25** | **3.72** | **3.25** |
| H16_B0690 | ABC-type RTX toxin transporter, ATPase and | **1.84** | ND | ND |
| H16_A2382 | ABC-type transporter, periplasmic component | **1.66** | 0.77 | 1.28 |
| H16_B2041 | ABC-type transporter, branched chain amino acids | **-2.07** | -0.39 | **-3.06** |
| H16_B0061 | ABC-type transporter, periplasmic component | **-3.24** | 0.9 | -1.17 |
| H16_A2879 | Cation/multidrug efflux system outer membrane | ND | ND | **-2.73** |
| H16_B0581 | Cation/multidrug efflux system outer membrane | 0.18 | **1.79** | **-2.51** |
| H16_B0583 | Cation/multidrug efflux system outer membrane | 0.28 | **1.64** | **-2.48** |
| H16_A0982 | Flp pilus assembly protein CpaC | **-3.36** | 0.61 | **-5.73** |
| H16_B2416 | IcmF like protein | **-1.64** | **-1.87** | ND |
| H16_B2428 | Clp ATP depenent protease | **-3.33** | **-2.96** | ND |
| H16-B2432 | Hcp like protein | **-2.63** | **-3.17** | ND |
